# A dual-prototype morphological topology evolution network for clinically oriented brain tumor MRI segmentation

**DOI:** 10.3389/fonc.2026.1885019

**Published:** 2026-07-01

**Authors:** Yuejun Yao, Cai Jing, Xing Wang, Yao Liu, Tanjun Wei, Yi Liu, Xianhai Li

**Affiliations:** 1 Department of Neurosurgery, Dazhou Central Hospital, Dazhou, China; 2 Department of Pharmacy, Dazhou Integrated TCM & Western Medical Hospital, Dazhou, China

**Keywords:** brain tumor, clinical auxiliary diagnosis, magnetic resonance imaging, medical image segmentation, neuroimaging analysis

## Abstract

Automatic segmentation of brain tumor MRI images has important clinical significance for lesion localization, preoperative planning, and therapeutic efficacy assessment. To address the challenges of blurred boundaries, irregular morphology, and similar grayscale appearances between brain tumor regions and surrounding brain tissues, this study proposes a dual-prototype morphological evolution network. The proposed method adopts SegFormer as the backbone network and designs an adaptive dual-prototype representation module to enhance category discrimination through background and tumor prototypes. Meanwhile, a morphological evolution attention module is introduced to improve the model’s perception of irregular boundaries and local structures through differentiable soft morphological operations. Experimental results show that the proposed method achieves an mIoU of 0.8479, an mDice of 0.8937, an mRecall of 0.9141, and an HD95 of 23.47 on the public brain tumor dataset. On the clinical brain tumor dataset, the corresponding values reach 0.7937, 0.8422, 0.8673, and 29.86, respectively. These results validate the effectiveness of the proposed method in brain tumor region recognition, boundary localization, and clinical auxiliary segmentation.

## 1 Introduction

Brain tumors are among the intracranial diseases with high clinical risk in neurosurgery and medical imaging diagnosis and treatment. The lesion extent, spatial location, boundary morphology, and tissue infiltration characteristics directly affect surgical planning, radiotherapy target delineation, treatment response assessment, and long-term follow-up management. Magnetic resonance imaging has become an important imaging modality for clinical brain tumor evaluation because of its high soft-tissue resolution and its ability to clearly present brain tissue structures and tumor-related abnormal regions. In recent years, machine learning and deep learning techniques have been widely applied to brain tumor MRI image analysis and have shown considerable potential in lesion segmentation, tumor recognition, and computer-aided diagnosis and treatment (1–3). However, brain tumors usually exhibit obvious heterogeneity in clinical images, including large variations in tumor size, irregular morphology, blurred boundaries, heterogeneous local enhancement, and similar grayscale appearances to surrounding normal brain tissues. These characteristics make manual delineation time-consuming and susceptible to physician experience, image quality, and subjective judgment. Therefore, constructing an accurate, stable, and clinically applicable automatic brain tumor segmentation model is of great significance for reducing the burden of clinical image analysis, improving the consistency of lesion quantitative assessment, and assisting neurosurgical diagnosis and treatment decision-making (4, 5). Meanwhile, segmentation models intended for clinical application should also consider prediction errors, uncertainty, and reliability, so as to reduce the potential influence of incorrect segmentation on subsequent clinical judgment (6).

For automatic brain tumor MRI segmentation, existing studies have extensively explored multimodal fusion, three-dimensional modeling, attention mechanisms, Transformer architectures, and missing-modality learning. Yang et al. proposed D2-Net to address the problem of incomplete clinical multimodal MRI data, improving segmentation performance under missing-modality conditions through a dual disentanglement mechanism (7). Liu et al. combined pixel-level fusion and feature-level fusion to enhance the representation capability of tumor regions in multimodal MRI (8). Yang et al. further proposed a flexible fusion network to adapt to information complementarity and modality variations among different MRI modalities (9). In terms of spatial structure modeling, Jiang et al. introduced Swin Transformer into three-dimensional multimodal brain tumor segmentation to enhance the representation of long-range dependencies and spatial context (10). Cao et al. constructed a three-dimensional convolutional network with multi-branch attention to improve the modeling capability of multi-scale tumor structures in MRI images (11). Ting et al. proposed a multimodal Transformer for incomplete MRI data, further alleviating the missing-modality problem in real clinical scenarios (12). In addition, Liu et al. combined a spatial pyramid module with an attention mechanism for automatic glioblastoma segmentation to enhance local region recognition (13). Yadav et al. proposed EffUNet++ based on FLAIR MRI images, demonstrating the continuous improvement value of U-Net-based architectures in brain tumor segmentation (14). Although these methods have achieved promising results, limitations remain in low-contrast boundaries, morphologically irregular regions, local small structures, false positives in background tissues, and cross-distribution generalization. These problems become more pronounced in clinical MRI data, where complex variations in scanning conditions, tumor morphology, and tissue background may further weaken the stability of segmentation results.

To address the above problems, this study proposes a dual-prototype morphological evolution network for binary segmentation of brain tumor MRI images. Existing studies have shown that multi-scale contextual aggregation, attention fusion, and generative structural modeling can improve global semantic representation and local detail recovery in brain tumor segmentation (15, 16). Meanwhile, the global modeling advantages of Transformer architectures in brain tumor MRI segmentation have received extensive attention, but their support for fine-grained boundaries and clinical generalization still needs to be further strengthened (17). Region-aware and sequence information-guided multi-decoder designs also indicate that explicit modeling of tumor region structural characteristics can improve segmentation robustness in complex clinical scenarios (18). Based on these observations, this study adopts the SegFormer encoder as the basic feature extraction structure and uses multi-scale hierarchical features to simultaneously capture shallow textures, local edges, mid-level structures, and deep semantic information. On this basis, an adaptive dualprototype representation module is designed to construct category prototypes for background and tumor regions, respectively. Prototype responses and category contextual fusion are then used to enhance the matching relationship between pixel features and tumor semantics, thereby alleviating semantic confusion between normal brain tissues and tumor tissues. Furthermore, a morphological evolution attention module is designed to introduce differentiable morphological modeling into the feature space. Soft dilation, soft erosion, soft opening, and soft closing operations are used to simulate the expansion, contraction, local denoising, and structural completion processes of tumor regions. Different morphological branches are dynamically fused through an adaptive gating mechanism to enhance the model’s perception of irregular boundaries, local small structures, and structural continuity.

The main contributions of this study are summarized as follows:

- A dual-prototype morphological evolution network is proposed for binary segmentation of brain tumor MRI images. It combines dual-category prototype calibration and feature-space morphological evolution in a unified multi-scale framework. This design strengthens both tumor semantic discrimination and boundary structure modeling.
- An adaptive dual-prototype representation module is designed to characterize background and tumor feature centers. Unlike single-prototype calibration, this module uses dual prototype groups and their response differences to guide feature refinement. It improves the separation between tumor regions and similar brain tissues.
- A morphological evolution attention module is designed to model blurred boundaries, irregular shapes, and local structural variations. It introduces differentiable soft dilation, erosion, opening, and closing into the feature space. A gated fusion strategy is further used to adaptively select suitable morphological responses.
- Systematic experiments are conducted on both a public brain tumor dataset and a clinical brain tumor MRI dataset. The results confirm the effectiveness of the proposed modules in region overlap, pixel-level recognition, and boundary localization. They also support its potential value for clinical auxiliary brain tumor segmentation.

## 2 Data and methods

### 2.1 Figshare brain tumor dataset

#### 2.1.1 Dataset introduction

This study adopts the Figshare Brain Tumor Dataset to experimentally validate the proposed brain tumor segmentation method. The dataset was publicly released by Cheng et al. and contains 3064 T1-weighted contrast-enhanced magnetic resonance images from 233 patients, covering three common types of brain tumors, namely meningioma, glioma, and pituitary tumor. Different from datasets that only provide image-level category annotations, the Figshare Brain Tumor Dataset also provides tumor boundary coordinates and binary tumor masks, which enables pixel-level segmentation research for brain tumor regions. Each sample is stored in MATLAB.mat format and mainly contains image data, patient ID, tumor category label, tumor boundary points, and tumor region mask. In the experiments of this study, the image region is divided into background and tumor regions, where non-tumor pixels are regarded as the background class and the regions corresponding to the tumor mask are regarded as the target class. The basic information of this dataset is shown in **Table 1**.

**Table 1:** Basic information of the Figshare brain tumor dataset.

Item	Description
Dataset name	Figshare Brain Tumor Dataset
Image modality	T1-weighted contrast-enhanced MRI images
Number of samples	3064 two-dimensional MRI slices
Number of patients	233 patients
Tumor categories	Meningioma, glioma, and pituitary tumor
Class distribution	708 meningioma images, 1426 glioma images, and 930 pituitary tumor images
Data format	MATLAB.mat files
Annotation information	Image-level category labels, tumor boundary coordinates, and binary tumor masks
Segmentation setting	Binary pixel-level segmentation of background and tumor regions

#### 2.1.2 Dataset preprocessing

**Figure 1:**
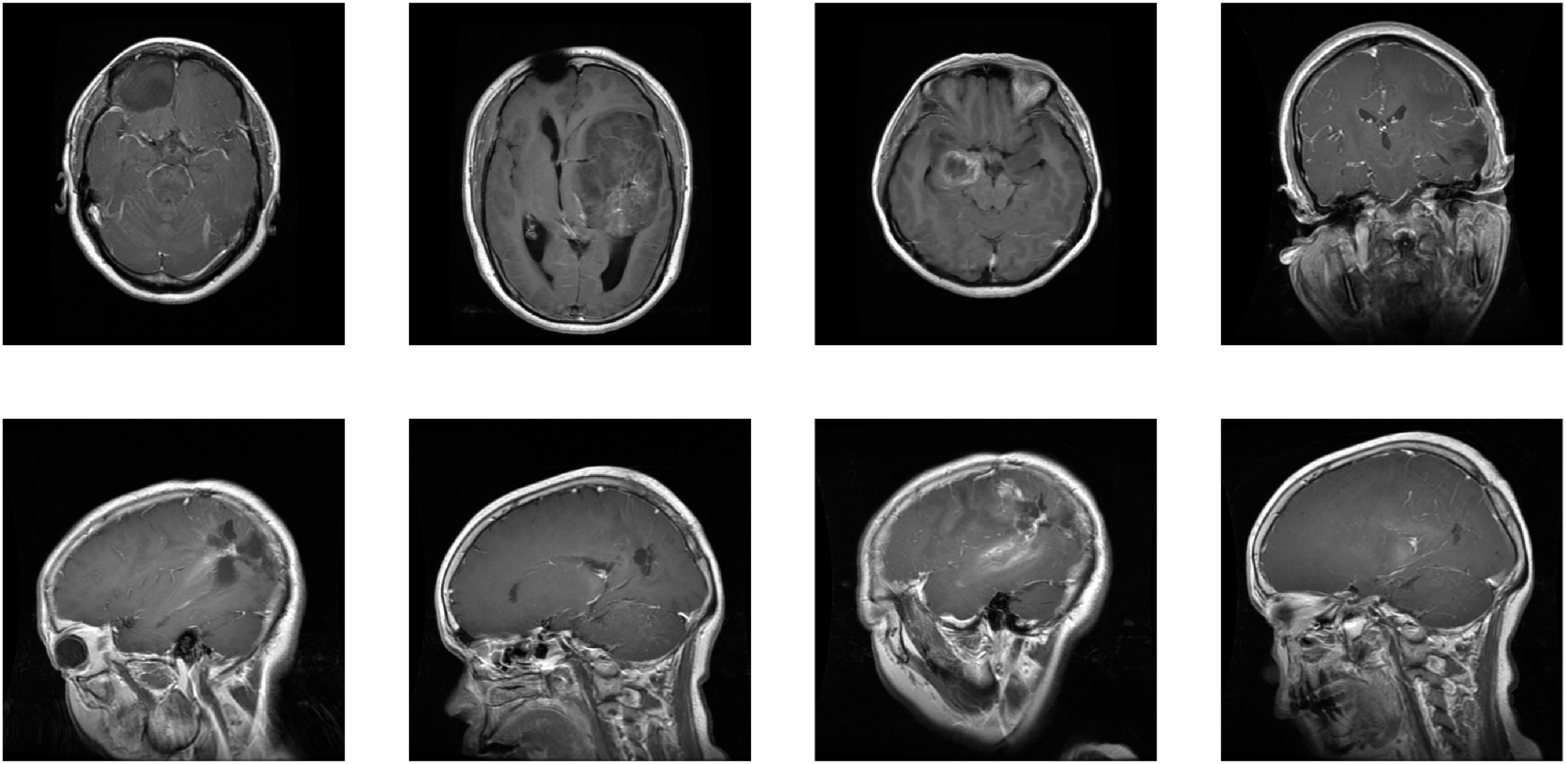
Public dataset examples.

To ensure the independence of experimental data partitioning and the consistency of segmentation annotations, this study first performs unified preprocessing on the original.mat files in the Figshare Brain Tumor Dataset. Specifically, the preprocessing script reads the cjdata field from each sample and extracts information including the patient ID, tumor category label, MRI image, tumor boundary coordinates, and tumor mask. Since some files in this dataset are stored in MATLAB v7.3 format, the script is compatible with both scipy.io and h5py reading methods, thereby avoiding sample loading failures caused by differences in file formats. During image processing, the original grayscale MRI images are converted into uint8 format. If the pixel values are already within the range of [0,1], they are directly linearly mapped to [0,255]; otherwise, intensity clipping and normalization are performed based on the 0.5% and 99.5% percentiles to reduce the influence of abnormal grayscale values on the dynamic range of the image. During label processing, although the original dataset provides three tumor categories, namely meningioma, glioma, and pituitary tumor, the research task in this study is not tumor type classification, but pixel-level segmentation of brain tumor regions. Therefore, the three tumor labels are only used for patient-level stratified partitioning and sample organization, while the actual segmentation labels are uniformly converted into a binary form, namely background region and tumor region. Specifically, if the tumorMask field exists in a sample, all pixels greater than 0 are assigned as tumor foreground, while the remaining pixels are assigned as background. If the tumorMask field is missing, a closed polygon mask is generated according to the tumor boundary coordinates provided by tumorBorder, and the region inside the polygon is regarded as the tumor foreground. In the final binary mask, background pixels are assigned a value of 0, and tumor pixels are assigned a value of 255, thereby forming standard binary segmentation annotations. For data partitioning, this study divides the Figshare Brain Tumor Dataset into training, validation, and test sets at the patient level with a ratio of 8:1:1. Specifically, 186 patients were used for training, 23 patients were used for validation, and 24 patients were used for testing. The validation set was used only for hyperparameter selection and model checkpoint selection, while the test set was kept independent and was not used for parameter tuning. During the partitioning process, stratified sampling is performed according to the tumor category of each patient to maintain consistent category distributions across different subsets as much as possible. Compared with direct random partitioning based on image slices, patient-level partitioning can prevent different slices from the same patient from appearing simultaneously in the training and test sets, thereby reducing the risk of data leakage. Examples from the dataset are shown in **Figure 1**.

### 2.2 Clinical dataset

This study further constructed a clinical brain tumor MRI dataset from Dazhou Integrated Traditional Chinese and Western Medicine Hospital to evaluate the generalization performance and application stability of the proposed method in real clinical scenarios. The clinical dataset contains retrospective imaging data from 137 patients with brain tumors. The collected images mainly consisted of contrast-enhanced T1-weighted brain MRI slices acquired during routine clinical examinations, and the dataset included cases with visible tumor regions and available manual segmentation annotations. Cases with severe motion artifacts, incomplete imaging information, poor image quality, or ambiguous lesion regions that were difficult to delineate were excluded from this study. All cases were anonymized and de-identified before data organization and experimental analysis, and personally identifiable information, including patient names, hospitalization numbers, and examination numbers, was removed. According to local laws, regulations, and ethical review requirements, this study was a retrospective medical imaging analysis that involved no additional clinical intervention, and all research data had been deidentified; therefore, renewed informed consent from patients was not required. This study was approved by the Medical Ethics Committee of Dazhou Integrated Traditional Chinese and Western Medicine Hospital, with the ethics approval number 2023 Review No. 10. The research process strictly followed the basic ethical principles for medical research stated in the Declaration of Helsinki. For manual annotation, tumor regions were delineated on MRI slices by clinicians with experience in brain tumor imaging interpretation, and the annotations were further reviewed to reduce obvious boundary inconsistency and improve label reliability. When disagreement or uncertainty occurred during annotation checking, the final mask was determined through discussion and adjudication based on the visible lesion boundary and clinical imaging information. During experimental partitioning, all samples were strictly divided into training and testing sets at the case level to prevent different image slices from the same patient from appearing simultaneously in the training and testing sets, thereby reducing the risk of data leakage. Finally, the dataset was divided into training and testing sets at a ratio of 8:2 for model training and clinical generalization performance evaluation. Specifically, 110 cases were used for training and 27 cases were used for testing. The main hyperparameters were determined based on the validation set of the public dataset and then kept fixed for the clinical dataset experiments, and no additional hyperparameter tuning was performed on the clinical test set. The exact patient-level and case-level partitioning information for both datasets is summarized in **Table 2**. The basic information of the dataset is shown in **Table 3**.

**Table 2:** Detailed data partitioning of the public and clinical brain tumor MRI datasets.

Dataset	Subset	Patients/cases	Usage
Figshare Brain Tumor Dataset	Training set	186 patients	Model training
Figshare Brain Tumor Dataset	Validation set	23 patients	Hyperparameter and checkpoint selection
Figshare Brain Tumor Dataset	Test set	24 patients	Final independent testing
Clinical Brain Tumor Dataset	Training set	110 cases	Model training
Clinical Brain Tumor Dataset	Test set	27 cases	Clinical generalization evaluation

**Table 3:** Basic information of the clinical brain tumor MRI dataset.

Item	Description
Data type	Retrospective clinical brain tumor MRI imaging data
Number of cases	137 patients with brain tumors
Study type	Retrospective medical imaging analysis study
Ethics approval institution	Medical Ethics Committee of Dazhou Integrated Traditional Chinese and Western Medicine Hospital
Ethics approval number	2023 Review No. 10
Ethical principle	Followed the basic ethical principles of the Declaration of Helsinki for medical research
Informed consent	Waived according to local laws, regulations, and ethical requirements for retrospective de-identified data
Privacy protection	Removed names, hospitalization numbers, examination numbers, and other personally identifiable information
Data partitioning strategy	Training and testing sets were divided at the case level
Training/testing ratio	8:2
Segmentation task setting	Binary pixel-level segmentation of brain tumor regions

### 2.3 Method

#### 2.3.1 Overall model architecture

**Figure 2:**
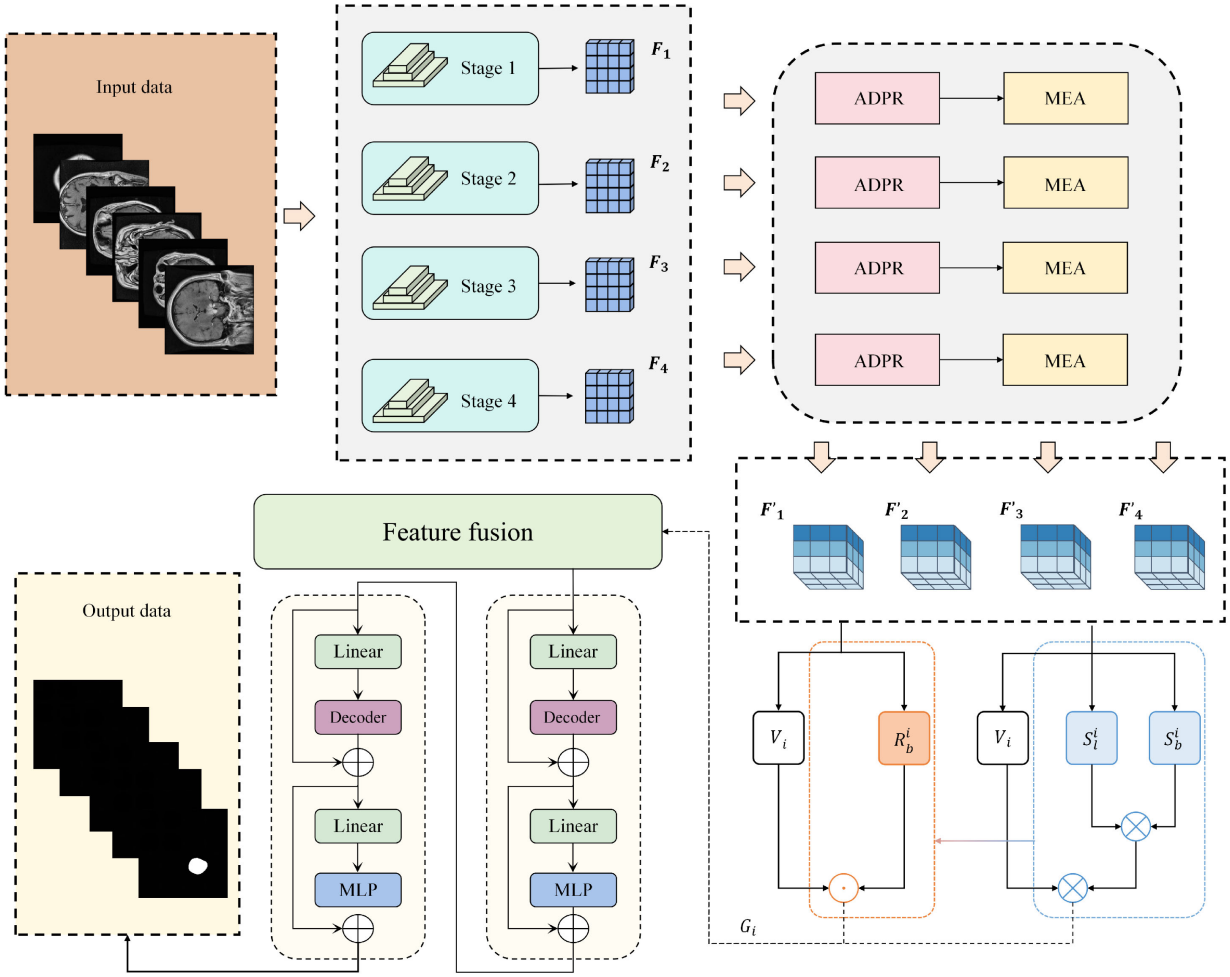
Overall architecture of the proposed brain tumor segmentation network.

This paper constructs a dual-prototype morphological evolution network for binary brain tumor segmentation in MRI images. The overall architecture consists of a multi-stage feature encoder, a semantic prototype calibration module, a morphological evolution module, and a multi-scale decoding fusion module. Different from existing prototype-based segmentation methods that usually rely on a single class center or directly use prototype similarity for pixel classification, the proposed Adaptive Dual Prototype Representation (ADPR) explicitly maintains background and tumor prototype groups at each feature scale and uses their response difference to guide category-aware contextual calibration. This design enables the model to simultaneously enhance tumor-related semantic responses and suppress background-tissue interference in low-contrast MRI regions. In addition, different from conventional morphologyinspired operations that are commonly applied to binary masks or used as fixed post-processing constraints, the proposed Morphological Evolution Attention (MEA) introduces differentiable soft dilation, soft erosion, soft opening, and soft closing into the intermediate feature space, and further learns adaptive branch weights through a gated fusion mechanism. Therefore, the proposed framework does not simply combine prototype learning and morphological operations, but integrates dual-category prototype response modeling with sample-adaptive morphological feature evolution for joint semantic discrimination and boundary-structure refinement. Given an input MRI image $X\in {\mathbb{R}}^{H\times W\times 1}$, the encoder first progressively extracts hierarchical features at four scales from shallow textures, local edges, intermediate structures, and deep semantics, obtaining the feature set {*F*_1_*,F*_2_*,F*_3_*,F*_4_}. Among them, shallow features preserve more spatial details and tumor boundary information, while deep features contain stronger global context and category-level semantic information. To avoid the limitation that single-scale features are difficult to simultaneously describe tumor boundary details and high-level semantics, a unified feature enhancement path is introduced after each encoding stage, enabling features at different resolutions to receive dual-prototype response calibration and adaptive morphological evolution enhancement. The overall encoding process can be formulated as shown in Equation 1:

(1) $${\left\{F_{i}\right\}}_{i=1}^{4}=ℰ\left(X\right),\;F_{i}\in {\mathbb{R}}^{\frac{H}{s_{i}}\times \frac{W}{s_{i}}\times C_{i}},\;s_{i}\in \left\{4,8,16,32\right\},$$

where ℰ(·) denotes the multi-stage encoder, *F_i_* denotes the feature map output by the *i*-th stage, *s_i_* denotes the downsampling ratio of the corresponding stage, and *C_i_* denotes the number of feature channels. **Figure 2** illustrates the overall structure of the proposed model.

In the multi-scale feature enhancement stage, each level of encoded features is first fed into the adaptive dual-prototype calibration module to model the category prototype responses of the background region and tumor region, respectively, thereby alleviating semantic confusion between brain tissues and tumor areas. Subsequently, the prototype-calibrated features are further input into the morphological evolution module, where soft dilation, soft erosion, soft opening, and soft closing operations are used to simulate various morphological changes of tumor regions. An adaptive gating mechanism is then employed to enhance the representation capability of irregular boundaries, small structures, and local structural regions. The enhanced four-level features are denoted as $\left\{F_{1}^{\prime },F_{2}^{\prime },F_{3}^{\prime },F_{4}^{\prime }\right\}$ and are fed into the multi-scale fusion decoder for scale alignment, contextual fusion, and spatial resolution recovery. Finally, the segmentation probability map of background and tumor pixels is generated. The overall prediction process can be formulated as shown in Equation 2:

(2) $$\hat{Y}=D\left(\text{Fuse }\left({\left\{U_{i}\left({ℳ}_{i}\left(P_{i}\left(F_{i}\right)\right)\right)\right\}}_{i=1}^{4}\right)\right),$$

where $P_{i}\left(\cdot \right)$ denotes the adaptive dual-prototype calibration module at the *i*-th scale, ${ℳ}_{i}\left(\cdot \right)$ denotes the morphological evolution module, $U_{i}\left(\cdot \right)$ denotes the scale alignment operation, Fuse(·) denotes multi-scale feature fusion, D(·) denotes the segmentation decoder, and $\hat{Y}$ denotes the final binary segmentation result of brain tumors.

#### 2.3.2 Adaptive dual prototype representation

**Figure 3:**
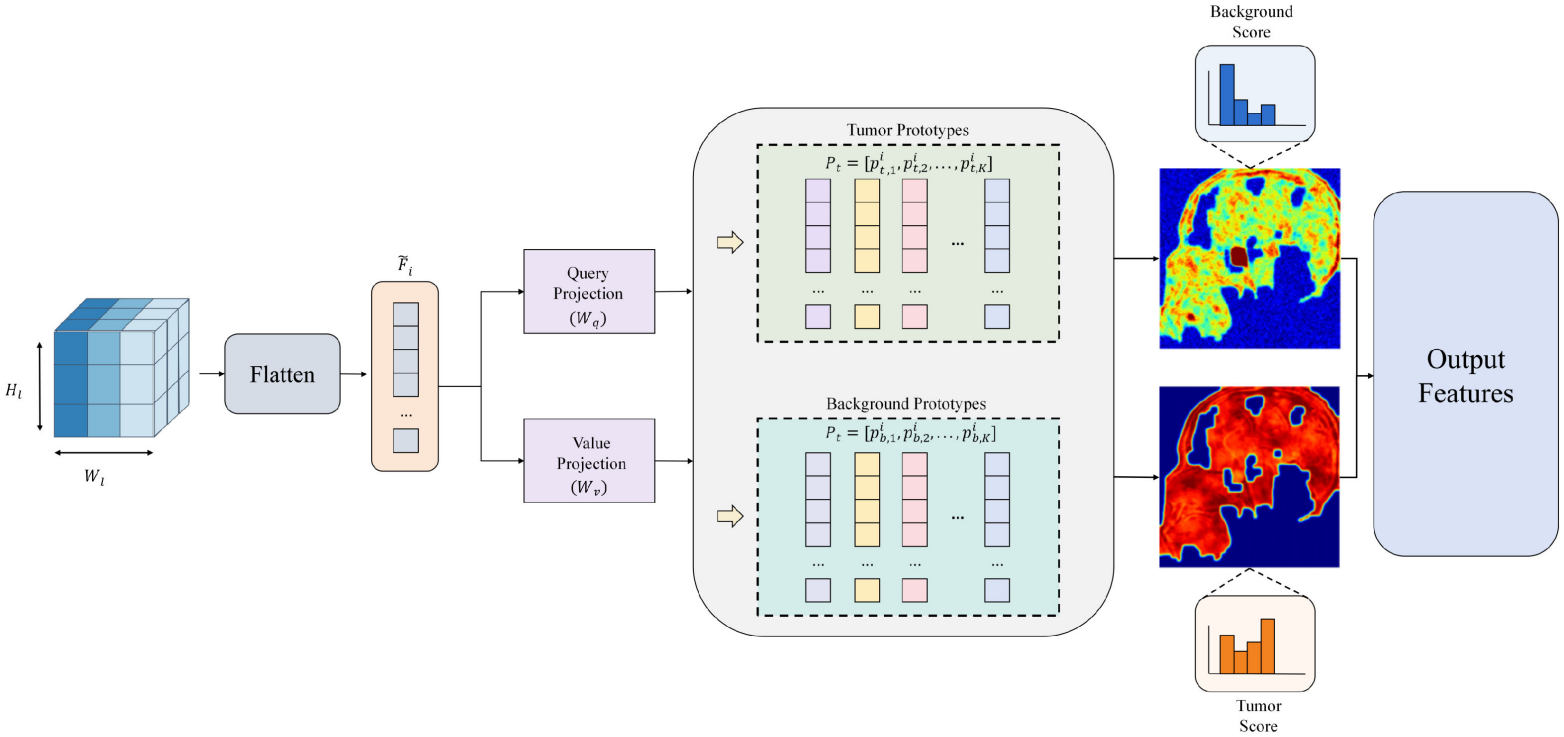
Architecture of the proposed adaptive dual prototype representation module.

The main difficulty in brain tumor MRI segmentation lies in the strong similarity between tumor regions and surrounding brain tissues in terms of grayscale intensity, local texture, and boundary transition. Relying solely on pixel-level local features may easily cause background tissues to be misclassified as tumor regions or lead to discontinuities in low-contrast tumor boundaries. To enhance the category discrimination capability of the model for background and tumor regions, this paper designs an Adaptive Dual Prototype Representation (ADPR) module. By explicitly constructing background prototypes and tumor prototypes, pixel features are mapped into the category prototype space, and prototype responses are used to perform contextual calibration on the original features. Given the input feature of the *i*-th scale $F_{i}\in {\mathbb{R}}^{H_{i}\times W_{i}\times C_{i}}$, it is first flattened into a pixel sequence, and query projection and value projection are then applied to obtain feature representations for prototype matching and contextual reconstruction, respectively. As shown in Equation 3:

(3) $${\tilde{F}}_{i}=\text{Reshape }(F_{i})\in {\mathbb{R}}^{N_{i}\times C_{i}},\;Q_{i}=\text{LN }({\tilde{F}}_{i})W_{q}^{i},\;V_{i}=\text{LN }({\tilde{F}}_{i})W_{v}^{i}$$

where $N_{i}=H_{i}W_{i}$ denotes the number of pixel locations at the *i*-th scale, $W_{q}^{i}\in {\mathbb{R}}^{C_{i}\times d_{i}}$ and $W_{v}^{i}\in {\mathbb{R}}^{C_{i}\times d_{i}}$ denote the query projection matrix and value projection matrix, respectively. *Q_i_* is used to measure the correlation between pixel features and category prototypes, while *V_i_* is used to preserve local detail information in the original features. The structure of ADPR is shown in **Figure 3**.

To separately characterize the category centers of non-tumor brain tissues and tumor regions, ADPR maintains two groups of learnable prototypes at each scale, namely background prototypes $P_{b}^{i}$ and tumor prototypes $P_{t}^{i}$. Different from a single category center, the multi-prototype representation can describe intra-class structural variations, such as the appearance differences among gray matter, white matter, and cerebrospinal fluid in background regions, as well as the feature variations among strongly enhanced, weakly enhanced, and boundary transition areas in tumor regions. The dual-prototype set at the *i*-th scale is defined as shown in Equation 4:

(4) $$P_{i}=\left\{P_{b}^{i},P_{t}^{i}\right\},\;P_{c}^{i}={\left[p_{c,1}^{i},p_{c,2}^{i},\cdots ,p_{c,K}^{i}\right]}^{⊤}\in {\mathbb{R}}^{K\times d_{i}},\;c\in \left\{b,t\right\}$$

where *K* denotes the number of prototypes for each category, and $p_{c,K}^{i}$ denotes the *k*-th prototype vector of category *c*. To improve the stability of prototype matching, the query features and category prototypes are normalized, and the response relationship between each pixel location and different category prototypes is computed based on scaled dot-product similarity as shown in Equation 5:

(5) $$A_{c}^{i}=\text{Softmax }\left(\frac{\text{Norm}\;(Q_{i})\text{Norm}\;{\left(P_{c}^{i}\right)}^{⊤}}{\tau_{i}\sqrt{d_{i}}}\right),\;R_{c}^{i}=A_{c}^{i}P_{c}^{i},\;c\in \left\{b,t\right\}$$

where $A_{c}^{i}\in {\mathbb{R}}^{N_{i}\times K}$ denotes the attention response of pixel features to the prototypes of category *c*, τ*_i_* denotes the temperature coefficient used to adjust the smoothness of prototype responses, and $R_{c}^{i}\in {\mathbb{R}}^{N_{i}mesd_{i}}$ denotes the category context representation aggregated from category prototypes. It should be noted that the Softmax operation in **Equation 5** is performed within the *K* prototypes of each category, and is used to obtain the intra-category prototype aggregation weights rather than the final background–tumor classification probability.

After obtaining the background prototype response and tumor prototype response, ADPR further generates category-aware response score maps to estimate whether each pixel location is closer to the background category or the tumor category. This process not only depends on the query features and prototype context themselves, but also introduces element-wise interaction terms between them to enhance the matching discriminability between pixel features and category prototypes. The response scores of the background and tumor categories can be expressed as shown in Equation 6:

(6) $$S_{c}^{i}=\text{Reshape }\left[\sigma \left(\phi_{s}^{i}\left(\left[Q_{i},\;R_{c}^{i},\;Q_{i}⊙R_{c}^{i},\;\left|Q_{i}-R_{c}^{i}\right|\right]\right)\right)\right],\;c\in \left\{b,t\right\}$$

where $S_{c}^{i}\in {\mathbb{R}}^{H_{i}\times W_{i}\times 1}$ denotes the spatial response score map of category *c*, $\phi_{s}^{i}\left(\cdot \right)$ denotes the score prediction function composed of linear mapping and nonlinear activation, ⊙ denotes element-wise multiplication, and σ(·) denotes the Sigmoid activation function. In implementation, the same score prediction function $\phi_{s}^{i}\left(\cdot \right)$ is applied to the background and tumor prototype contexts, so that the two response maps are generated under a shared matching criterion. Specifically, *Q_i_* and $R_{c}^{i}$ have the same dimension of ${\mathbb{R}}^{N_{i}\times d_{i}}$, and their concatenation with the interaction terms produces a feature of ${\mathbb{R}}^{N_{i}\times 4d_{i}}$, which is mapped to ${\mathbb{R}}^{N_{i}s1}$ and then reshaped into $H_{i}\times W_{i}\times 1$. The response maps $S_{b}^{i}$ and $S_{t}^{i}$ are not directly used as mutually exclusive final segmentation probabilities. Instead, their relative difference is further used in the subsequent adaptive fusion process, and the final mutually exclusive background–tumor prediction is generated by the segmentation decoder under pixel-level supervision. Through the joint modeling of $S_{b}^{i}$ and $S_{t}^{i}$, the module can highlight tumor-related regions in the feature space while suppressing interfering regions highly correlated with background prototypes, thereby alleviating semantic confusion between brain tissues and tumor boundaries.

To achieve more flexible prototype context injection, ADPR designs a prototype context fusion mechanism that adaptively allocates the contributions of background context and tumor context according to the category response differences at pixel locations. Specifically, the module first generates fusion gating weights based on the original value features, background prototype context, tumor prototype context, and the difference relationship between them as shown in Equation 7:

(7) $$G_{i}=\sigma \left(\phi_{g}^{i}\left(\left[V_{i},\;R_{b}^{i},\;R_{t}^{i},\;R_{t}^{i}-R_{b}^{i},\;\text{Vec }(S_{t}^{i})-\text{Vec }(S_{b}^{i})\right]\right)\right)$$

where $G_{i}\in {\mathbb{R}}^{N_{i}\times d_{i}}$ denotes the adaptive fusion gate, $\phi_{g}^{i}\left(\cdot \right)$ denotes the gate generation function, and Vec(·) denotes the operation of flattening the spatial score map back into a pixel sequence. Finally, the module performs residual fusion between the dual-prototype context and the original value features to obtain enhanced features calibrated by category semantics as shown in Equation 8:

(8) $$F_{i}^{\text{ADPR }}=F_{i}+\gamma_{i}\cdot {\text{Reshape}}^{-1}\left\{\phi_{o}^{i}\left[V_{i}+G_{i}⊙R_{t}^{i}+\left(1-G_{i}\right)⊙R_{b}^{i}\right]\right\}$$

where $\phi_{o}^{i}\left(\cdot \right)$ denotes the output mapping function, γ*_i_* denotes the learnable residual coefficient, and $F_{i}^{\text{ADPR }}$ denotes the feature of the *i*-th scale enhanced by the Adaptive Dual Prototype Representation module. This design enables the model to introduce category-level prototype priors while preserving original spatial details, thereby enhancing the semantic consistency of tumor regions and the suppression capability for background regions, and providing more stable discriminative feature inputs for the subsequent morphological evolution module.

#### 2.3.3 Morphological evolution attention

**Figure 4:**
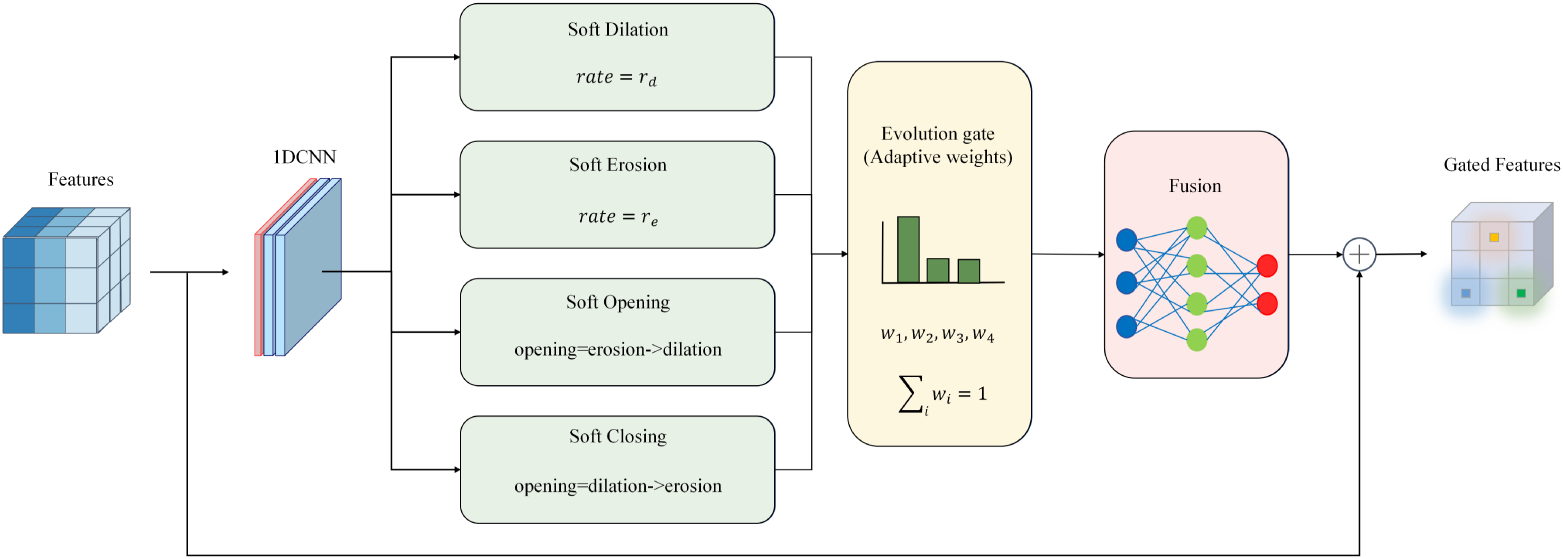
Architecture of the proposed Morphological Evolution Attention module.

Brain tumor regions usually exhibit irregular morphology, blurred boundaries, local protrusions, internal cavities, and obvious scale variations. A single convolutional receptive field is difficult to fully describe the spatial topological continuity and morphological evolution relationships of tumor regions. To this end, this paper designs a Morphological Evolution Attention (MEA) module, which introduces a differentiable morphological modeling mechanism into the feature space. Soft dilation, soft erosion, soft opening, and soft closing operations are used to characterize the expansion tendency, contraction tendency, small-noise suppression capability, and local cavity completion capability of tumor regions, respectively. Given the ADPR-calibrated feature at the *i*-th scale $F_{i}^{\text{ADPR }}\in {\mathbb{R}}^{H_{i}\times W_{i}\times C_{i}}$, MEA first obtains the morphological evolution base feature through channel interaction mapping as shown in Equation 9:

(9) $$Z_{i}={\text{Reshape}}^{-1}\left(\phi_{c}^{i}\left(\text{LN }\left(\text{Reshape }\left(F_{i}^{\text{ADPR }}\right)\right)\right)\right),\;Z_{i}\in {\mathbb{R}}^{H_{i}\times W_{i}\times C_{i}}$$

where $\phi_{c}^{i}\left(\cdot \right)$ denotes the channel interaction function composed of one-dimensional convolution and nonlinear activation, which is used to enhance the dependencies among different channels without destroying the spatial structure. The overall structure of MEA is shown in **Figure 4**.

In the morphological evolution branch, this paper adopts a smooth extremum approximation to construct differentiable soft morphological operators, enabling traditional morphological operations to be embedded into the end-to-end neural network training process. For a spatial position *p* and its neighborhood ${\text{Ω}}_{r}\left(p\right)$, soft dilation and soft erosion are respectively defined as:

(10) $$D_{r}\left(Z_{i}\right)\left(p,c\right)=\tau_{m}\text{log }\sum_{q\in {\text{Ω}}_{r}\left(p\right)}\text{exp }\left(\frac{Z_{i}\left(q,c\right)}{\tau_{m}}\right),\;{ℰ}_{r}\left(Z_{i}\right)\left(p,c\right)=-\tau_{m}\text{log }\sum_{q\in {\text{Ω}}_{r}\left(p\right)}\text{exp }\left(-\frac{Z_{i}\left(q,c\right)}{\tau_{m}}\right)$$

As shown in Equation 10, where $D_{r}\left(\cdot \right)$ and ${ℰ}_{r}\left(\cdot \right)$ denote the soft dilation and soft erosion operations with radius *r*, respectively, τ*_m_* is the morphological smoothing coefficient, and *c* denotes the channel index. Furthermore, soft opening and soft closing are obtained by sequentially combining soft erosion and soft dilation, which are used to describe the local denoising and structural completion processes of tumor morphology as shown in Equation 11:

(11) $$O_{r_{o}}\left(Z_{i}\right)=D_{r_{o}}\left({ℰ}_{r_{o}}\left(Z_{i}\right)\right),\;C_{r_{c}}\left(Z_{i}\right)={ℰ}_{r_{c}}\left(D_{r_{c}}\left(Z_{i}\right)\right)$$

where $O_{r_{o}}\left(\cdot \right)$ denotes the soft opening operation, which can suppress local isolated noise and pseudo responses, while $C_{r_{c}}\left(\cdot \right)$ denotes the soft closing operation, which can enhance boundary continuity and compensate for weak-response cavities inside tumor regions. The outputs of the four morphological branches can be uniformly expressed as shown in Equation 12:

(12) $$\left\{T_{i}^{d},T_{i}^{e},T_{i}^{o},T_{i}^{c}\right\}=\left\{\phi_{d}^{i}\left(D_{r_{d}}\left(Z_{i}\right)\right),\phi_{e}^{i}\left({ℰ}_{r_{e}}\left(Z_{i}\right)\right),\phi_{o}^{i}\left(O_{r_{o}}\left(Z_{i}\right)\right),\phi_{cl}^{i}\left(C_{r_{c}}\left(Z_{i}\right)\right)\right\}$$

where $T_{i}^{d},T_{i}^{e},T_{i}^{o},\text{ and }T_{i}^{c}$ denote the morphology-enhanced features from the dilation, erosion, opening, and closing branches, respectively. $\phi_{d}^{i}\left(\cdot \right),\phi_{e}^{i}\left(\cdot \right),\phi_{o}^{i}\left(\cdot \right),\text{ and }\phi_{cl}^{i}\left(\cdot \right)$ are the branch feature mapping functions.

Since different brain tumor samples exhibit significant differences in shape, size, and boundary clarity, fixed fusion of different morphological branches may weaken the model’s adaptability to sample-specific structures. Therefore, MEA further introduces a morphological evolution gating mechanism, which adaptively generates the weights of different morphological branches according to the global structural description of the current input feature. Specifically, global statistical aggregation is first performed on the morphological branch features, and normalized branch weights are then obtained through a gating mapping:

(13) $$\alpha_{i}=\text{Softmax }\left(\phi_{g}^{i}\left(\left[\text{GAP }(T_{i}^{d}),\text{GAP }(T_{i}^{e}),\text{GAP }(T_{i}^{o}),\text{GAP }(T_{i}^{c})\right]\right)\right),\;\sum_{k\in \left\{d,e,o,c\right\}}\alpha_{i}^{k}=1$$

As shown in Equation 13, where $\alpha_{i}=\left\{\alpha_{i}^{d},\alpha_{i}^{e},\alpha_{i}^{o},\alpha_{i}^{c}\right\}$ denotes the adaptive evolution weights of the four morphological branches, GAP(·) denotes global average pooling, and $\phi_{g}^{i}\left(\cdot \right)$ denotes the gating weight generation function. Finally, different morphological branches are adaptively fused under the constraint of gating weights, and the morphology-enhanced feature is output through a residual connection as shown in Equation 14:

(14) $$F_{i}^{\text{MEA }}=F_{i}^{\text{ADPR }}+\lambda_{i}\cdot \phi_{f}^{i}\left(\text{Concat }\left[Z_{i},\sum_{k\in \left\{d,e,o,c\right\}}\alpha_{i}^{k}T_{i}^{k},Z_{i}⊙\sum_{k\in \left\{d,e,o,c\right\}}\alpha_{i}^{k}T_{i}^{k}\right]\right)$$

where $\phi_{f}^{i}\left(\cdot \right)$ denotes the fusion mapping function, λ*_i_* is the learnable residual adjustment coefficient, and $F_{i}^{\text{MEA }}$ denotes the feature enhanced by Morphological Evolution Attention. This mechanism can dynamically select appropriate morphological evolution paths according to the tumor morphological characteristics of different samples. While preserving the original semantic representation, it strengthens the responses of tumor boundaries, local small structures, and irregular topological regions, thereby providing more stable structure-aware features for subsequent multi-scale fusion decoding.

## 3 Experimental setup and evaluation metric

### 3.1 Experimental setup

The experiments in this study are implemented based on the PyTorch deep learning framework. The backbone network adopts the SegFormer encoder, and the proposed ADPR and MEA modules are embedded after its multi-scale feature outputs to accomplish the binary segmentation task of brain tumor regions. Specifically, SegFormer-B2 is used as the backbone variant, and ImageNet-pretrained weights are adopted for encoder initialization. The decoder adopts a multi-scale feature fusion structure to integrate the enhanced features from different stages and generate the final binary segmentation map. All input MRI images and corresponding binary masks are uniformly resized to 512 × 512. The training, validation, and test sets are divided at the patient level to avoid data leakage caused by samples from the same patient appearing in different subsets. The model is trained for 200 epochs. AdamW is adopted as the optimizer, and a cosine annealing learning rate scheduling strategy is used to improve the stability of the training process. To reduce the influence of random initialization and data shuffling, each experiment is independently repeated three times with different random seeds, and the final results are reported as the mean and standard deviation over the three runs. The total loss is composed of Cross-Entropy Loss and Dice Loss with equal weights. The ADPR module uses dual category prototypes for background and tumor regions, while the MEA module contains four soft morphological branches, including dilation, erosion, opening, and closing. During training, data augmentation methods such as random flipping, random rotation, and intensity perturbation are applied to enhance the adaptability of the model to variations in tumor location, morphology, and grayscale distribution. The detailed experimental setup is shown in **Table 4**.

**Table 4:** Experimental environment and main hyperparameter settings.

Item	Setting
Operating system	Ubuntu 20.04
Programming language	Python 3.10
Deep learning framework	PyTorch 2.1
CUDA version	CUDA 11.8
GPU	NVIDIA RTX 4090D 24GB
Backbone network	SegFormer-B2
Pretrained weights	ImageNet-pretrained encoder weights
Decoder	Multi-scale feature fusion decoder
Input size	512 × 512
Number of segmentation classes	2 classes, background and tumor
Training epochs	200 epochs
Batch size	8
Optimizer	AdamW
Initial learning rate	1 × 10−4
Weight decay	1 × 10−4
Learning rate scheduling strategy	Cosine Annealing
Loss function	Cross-Entropy Loss + Dice Loss
Loss weights	1.0 for Cross-Entropy Loss and 1.0 for Dice Loss
ADPR setting	Dual prototypes for background and tumor categories
MEA kernel size	3 × 3 neighborhood for soft morphological operations
Morphological smoothing coefficient	τm = 1.0
MEA branch mapping	Convolutional mapping followed by nonlinear activation
Log-sum-exp stabilization	Maximum-value subtraction before exponential calculation
Morphological operation domain	Feature-space enhancement rather than binary mask post-processing

### 3.2 Evaluation metric

To comprehensively evaluate the region overlap, pixel classification, boundary localization, and overall prediction performance of the model in the binary brain tumor segmentation task, this study adopts mIoU, mDice, mPrecision, mRecall, PAcc, and HD95 as evaluation metrics. Let *C* denote the number of classes. In this study, *C* = 2, corresponding to the background class and tumor class, respectively. *TP_c_*, *FP_c_*, *FN_c_*, and *TN_c_* denote the numbers of true-positive, false-positive, false-negative, and true-negative pixels of the *c*-th class, respectively. *P* denotes the set of boundary points of the predicted segmentation region, and *G* denotes the set of boundary points of the ground-truth segmentation region.

mIoU is used to measure the mean intersection-over-union between the predicted regions and the ground-truth regions, which reflects the overall region overlap of the model across different classes as shown in Equation 15:

(15) $$\text{mIoU }=\frac{1}{C}\sum_{c=1}^{C}\frac{TP_{c}}{TP_{c}+FP_{c}+FN_{c}}$$

mDice is used to measure the mean Dice similarity coefficient between the predicted segmentation results and the ground-truth annotations, and it has strong representational ability for the overlap quality of target regions in medical images as shown in Equation 16:

(16) $$\text{mDice }=\frac{1}{C}\sum_{c=1}^{C}\frac{2TP_{c}}{2TP_{c}+FP_{c}+FN_{c}}$$

mPrecision is used to measure the proportion of correctly classified pixels among the pixels predicted as a certain class, which reflects the model’s ability to control false detections of tumor regions as shown in Equation 17:

(17) $$\text{mPrecision }=\frac{1}{C}\sum_{c=1}^{C}\frac{TP_{c}}{TP_{c}+FP_{c}}$$

mRecall is used to measure the proportion of pixels that truly belong to a certain class and are correctly identified by the model, which reflects the model’s sensitivity to missed detections of tumor regions as shown in Equation 18:

(18) $$\text{mRecall }=\frac{1}{C}\sum_{c=1}^{C}\frac{TP_{c}}{TP_{c}+FN_{c}}$$

PAcc is used to measure the proportion of correctly classified pixels among all pixels, which reflects the pixel-level classification accuracy of the model over the entire image:

(19) $$\text{PAcc }=\frac{\sum_{c=1}^{C}TP_{c}}{\sum_{c=1}^{C}(TP_{c}+FP_{c})}$$

As shown in Equation 19, HD95 is used to measure the 95th percentile Hausdorff distance between the predicted boundary and the ground-truth boundary, which reduces the influence of extreme outlier boundary points on boundary error evaluation as shown in Equation 20:

(20) $$\text{HD}95\;(P,G)=\text{max }\left\{Q_{95}\left(\left\{\underset{g\in G}{\min}\|p-g{\|}_{2}|p\in P\right\}\right),Q_{95}\left(\left\{\underset{p\in P}{\min}\|g-p{\|}_{2}|g\in G\right\}\right)\right\}$$

## 4 Experimental results and analysis

### 4.1 Experimental results compared with other models

To verify the effectiveness and generalization ability of the proposed method in the brain tumor segmentation task, this study conducts comparative experiments with several mainstream segmentation models on the Figshare Brain Tumor Dataset and a clinical brain tumor MRI dataset. For all comparative experiments, the same basic experimental settings were maintained, including data preprocessing, input size, batch size, optimizer configuration, training epochs, loss function, data augmentation strategy, and evaluation protocol, to ensure the fairness and consistency of model comparison. For SAM-based comparison methods, MedicoSAM was directly used for inference without additional training, because SAM itself is designed as a general segmentation foundation model. CertainTTA was not treated as an independent SAMbased model, but as a test-time adaptation inference framework; in our implementation, it adopted the same SAM model setting as MedicoSAM as the segmentation source model, and its final output was uniformly converted into the binary background–tumor mask format for evaluation. The comparative analysis is mainly carried out from three aspects, namely region overlap accuracy, pixel-level classification performance, and boundary localization error, to comprehensively evaluate the segmentation performance of different models on both the public dataset and real clinical data scenarios. The experimental results on the Figshare Brain Tumor Dataset are first presented in **Table 5**.

**Table 5:** Comparison of segmentation performance of different models on the brain tumor dataset.

Method	mIoU	mDice	mPrecision	mRecall	PAcc	HD95
U-Net Ronneberger et al. (19)	0.7018 ± 0.0060	0.7642 ± 0.0065	0.7817 ± 0.0036	0.7925 ± 0.0072	0.9861 ± 0.0024	39.82 ± 3.11
nnU-Net Isensee et al. (20)	0.7742 ± 0.0063	0.8295 ± 0.0156	0.8374 ± 0.0115	0.8508 ± 0.0175	0.9907 ± 0.0006	31.64 ± 1.22
DeepLabV3+ Chen et al. (21)	0.7926 ± 0.0184	0.8421 ± 0.0102	0.8312 ± 0.0132	0.8724 ± 0.0187	0.9911 ± 0.0017	30.27 ± 4.81
SegFormer Xie et al. (22)	0.8124 ± 0.0137	0.8586 ± 0.0119	0.8643 ± 0.0044	0.8791 ± 0.0198	0.9923 ± 0.0023	28.18 ± 3.99
S2S2 Pan et al. (23)	0.7315 ± 0.0210	0.7934 ± 0.0186	0.8107 ± 0.0043	0.8039 ± 0.0094	0.9876 ± 0.0037	36.75 ± 5.00
MSSM-MFP Zhang et al. (24)	0.8236 ± 0.0209	0.8668 ± 0.0108	0.8751 ± 0.0041	0.8836 ± 0.0197	0.9928 ± 0.0010	27.91 ± 3.73
CertainTTA Dong et al. (25)	0.7868 ± 0.0152	0.8362 ± 0.0089	0.8499 ± 0.0174	0.8435 ± 0.0159	0.9902 ± 0.0010	32.06 ± 2.20
Mask2Former Cheng et al. (26)	0.8312 ± 0.0136	0.8743 ± 0.0072	0.8805 ± 0.0128	0.8962 ± 0.0115	0.9931 ± 0.0033	26.84 ± 1.79
MedicoSAM Archit et al. (27)	0.8017 ± 0.0268	0.8519 ± 0.0110	0.8696 ± 0.0193	0.8597 ± 0.0124	0.9918 ± 0.0022	29.56 ± 3.10
HSMix Sun et al. (28)	0.8195 ± 0.0204	0.8641 ± 0.0126	0.8579 ± 0.0052	0.8948 ± 0.0047	0.9926 ± 0.0026	28.44 ± 3.14
PraNet-V2 Hu et al. (29)	0.8063 ± 0.0141	0.8548 ± 0.0230	0.8727 ± 0.0121	0.8612 ± 0.0168	0.9914 ± 0.0012	29.93 ± 2.31
Ours	0.8479 ± 0.0055	0.8937 ± 0.0113	0.8958 ± 0.0072	0.9141 ± 0.0034	0.9949 ± 0.0040	23.47 ± 2.46

**Table 5** presents the quantitative comparison results of different segmentation models on the Brain Tumor Dataset. It can be observed that the traditional U-Net performs relatively weakly across all metrics, with mIoU and mDice reaching only 0.7018 and 0.7642, respectively, indicating that a simple encoder–decoder structure is insufficient to fully model the complex morphological variations and blurred boundaries of brain tumor regions. Compared with UNet, nnU-Net, DeepLabV3+, and SegFormer all achieve obvious improvements. Among them, SegFormer obtains mIoU, mDice, and PAcc values of 0.8124, 0.8586, and 0.9923, respectively, demonstrating the advantage of the Transformer encoder in global contextual modeling. Furthermore, compared with advanced methods such as Mask2Former, MSSMMFP, and HSMix, the proposed method still achieves the best performance, reaching 0.8479, 0.8937, 0.8958, 0.9141, and 0.9949 in terms of mIoU, mDice, mPrecision, mRecall, and PAcc, respectively, while reducing HD95 to 23.47. Compared with Mask2Former, which shows the closest performance, the proposed method improves mIoU and mDice by 1.67% and 1.94%, respectively, and reduces HD95 by 3.37. These results indicate that the proposed ADPR and MEA can effectively enhance the category discrimination capability of tumor regions and the representation ability of boundary structures, thereby achieving more stable segmentation results in terms of region overlap accuracy, pixel-level recognition accuracy, and boundary localization error. Further experimental results using the clinical dataset are presented in **Table 6**.

**Table 6:** Comparison of segmentation performance of different models on the clinical brain tumor dataset.

Method	mIoU	mDice	mPrecision	mRecall	PAcc	HD95
U-Net	0.6419 ± 0.0217	0.7116 ± 0.0141	0.7358 ± 0.0050	0.7421 ± 0.0064	0.9828 ± 0.0032	47.32 ± 4.48
nnU-Net	0.7268 ± 0.0079	0.7924 ± 0.0090	0.8032 ± 0.0049	0.8175 ± 0.0204	0.9889 ± 0.0024	37.44 ± 1.33
DeepLabV3+	0.7149 ± 0.0076	0.7841 ± 0.0063	0.8297 ± 0.0175	0.7768 ± 0.0106	0.9875 ± 0.0020	39.06 ± 2.55
SegFormer	0.7453 ± 0.0171	0.8079 ± 0.0137	0.8115 ± 0.0188	0.8426 ± 0.0200	0.9897 ± 0.0033	35.28 ± 2.02
S2S2	0.6794 ± 0.0070	0.7427 ± 0.0031	0.7693 ± 0.0105	0.7618 ± 0.0119	0.9843 ± 0.0032	44.81 ± 4.86
MSSM-MFP	0.7628 ± 0.0067	0.8212 ± 0.0193	0.8469 ± 0.0099	0.8274 ± 0.0191	0.9906 ± 0.0039	34.52 ± 2.27
CertainTTA	0.7386 ± 0.0144	0.7985 ± 0.0209	0.7876 ± 0.0156	0.8492 ± 0.0223	0.9883 ± 0.0012	36.91 ± 4.66
Mask2Former	0.7814 ± 0.0035	0.8331 ± 0.0093	0.8517 ± 0.0205	0.8564 ± 0.0113	0.9910 ± 0.0009	32.38 ± 4.70
MedicoSAM	0.7567 ± 0.0144	0.8128 ± 0.0166	0.8684 ± 0.0191	0.8011 ± 0.0205	0.9898 ± 0.0015	35.97 ± 4.50
HSMix	0.7716 ± 0.0049	0.8264 ± 0.0208	0.8339 ± 0.0039	0.8645 ± 0.0171	0.9902 ± 0.0015	33.75 ± 3.12
PraNet-V2	0.7489 ± 0.0172	0.8061 ± 0.0186	0.8178 ± 0.0109	0.8316 ± 0.0167	0.9891 ± 0.0017	36.23 ± 2.98
Ours	0.7937 ± 0.0132	0.8422 ± 0.0091	0.8509 ± 0.0104	0.8673 ± 0.0130	0.9918 ± 0.0035	29.86 ± 1.62

**Figure 5:**
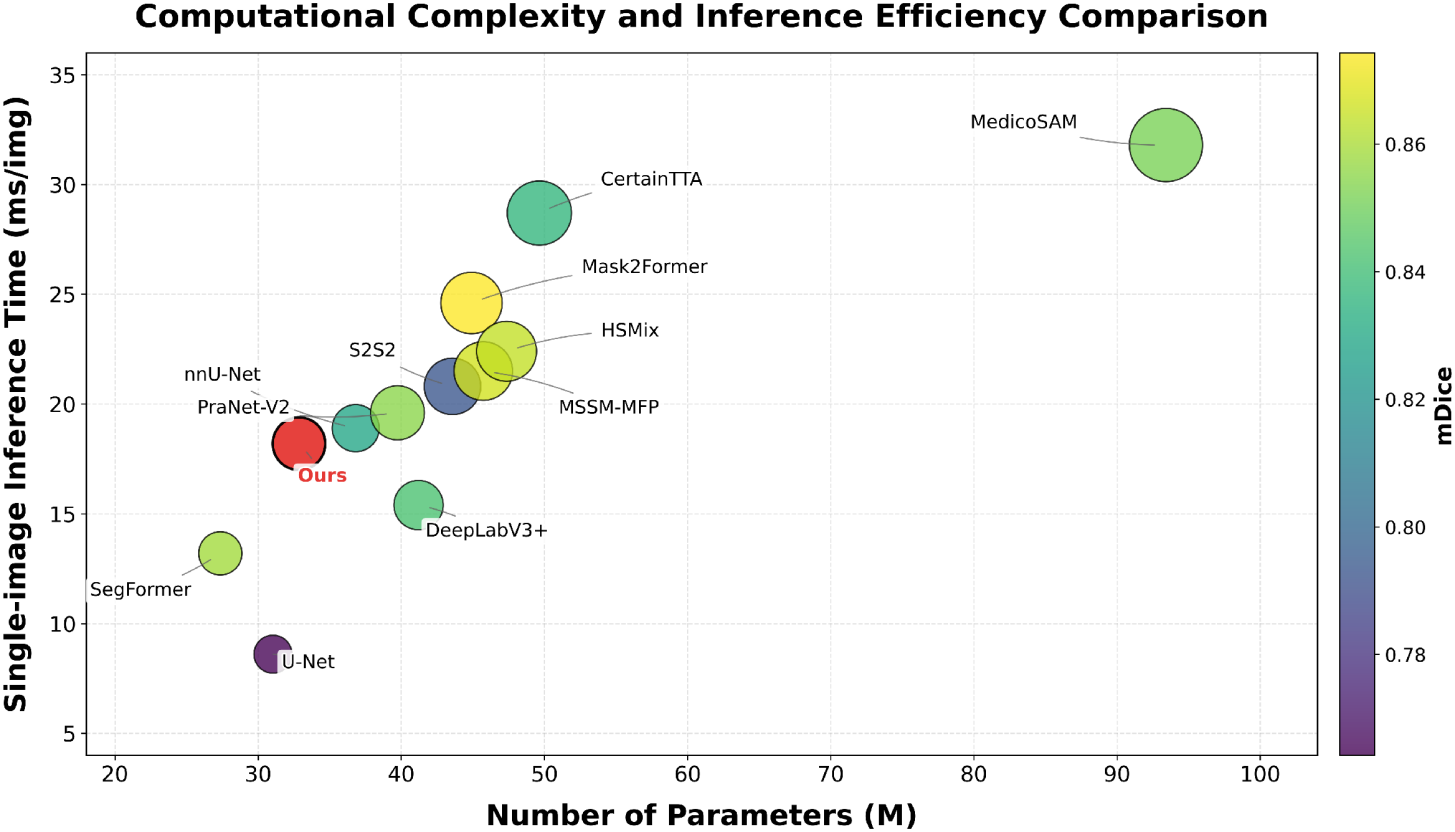
Bubble chart comparing model performance.

**Table 6** presents the segmentation performance of different models on the clinical brain tumor MRI dataset. Compared with the public dataset, clinical data usually suffer from differences in scanning conditions, larger variations in tumor scale, lower boundary contrast, and more complex tissue backgrounds. Therefore, the overall performance of all models shows a certain degree of degradation. Methods such as U-Net and S2S2 obtain relatively low mIoU and mDice values, indicating their limited adaptability to complex tumor structures in clinical scenarios. SegFormer achieves better results than traditional convolutional models, but its HD95 remains 35.28, suggesting that relying solely on global semantic modeling is still insufficient to effectively constrain tumor boundaries. Mask2Former and HSMix show strong performance on most metrics, while the proposed method achieves 0.7937, 0.8422, 0.8673, 0.9918, and 29.86 in terms of mIoU, mDice, mRecall, PAcc, and HD95, respectively, obtaining the best or near-best overall performance. In particular, in terms of HD95, the proposed method reduces the value by 2.52 compared with Mask2Former and by 3.89 compared with HSMix, indicating that the proposed MEA can enhance the representation ability of irregular boundaries and local structures through morphological evolution, thereby improving boundary deviation problems in clinical images. Meanwhile, the proposed method achieves higher mRecall than all comparison methods, demonstrating that ADPR can enhance the semantic response of tumor regions through adaptive representation of background prototypes and tumor prototypes, reducing missed segmentation in low-contrast regions. Although MedicoSAM achieves slightly higher mPrecision than the proposed method, its mRecall and HD95 are obviously weaker, suggesting that its prediction results tend to be more conservative and may sacrifice the integrity of tumor regions. Overall, the joint design of ADPR and MEA not only improves the model’s category discrimination capability for tumor regions, but also enhances its structure-aware ability for complex clinical morphological boundaries, thereby showing better stability and generalization ability on the real clinical dataset. Finally, we present the performance bubble chart for each model, as shown in **Figure 5**.

### 4.2 Ablation test results

To further verify the effectiveness of the proposed ADPR and MEA modules in the brain tumor segmentation task, this study designs ablation experiments under the same experimental settings. The ablation experiments use SegFormer as the baseline model and introduce different module combinations to analyze the influence of semantic prototype calibration and morphological evolution on model performance. By comparing different model variants in terms of region overlap, pixel recognition, and boundary localization metrics, the contribution of each component to the overall segmentation performance can be more intuitively evaluated. The experimental results are shown in **Table 7**.

**Table 7:** Ablation results of ADPR and MEA on two brain tumor segmentation datasets.

Method	mIoU	mDice	mPrecision	mRecall	PAcc	HD95
Brain tumor dataset						
SegFormer	0.8124 ± 0.0137	0.8586 ± 0.0119	0.8643 ± 0.0044	0.8791 ± 0.0198	0.9923 ± 0.0023	28.18 ± 3.99
SegFormer + ADPR	0.8326 ± 0.0098	0.8782 ± 0.0146	0.8867 ± 0.0061	0.8983 ± 0.0125	0.9936 ± 0.0018	25.96 ± 3.31
SegFormer + MEA	0.8279 ± 0.0152	0.8715 ± 0.0068	0.8764 ± 0.0133	0.9027 ± 0.0059	0.9932 ± 0.0047	26.74 ± 1.88
Ours w/Single-prototype ADPR	0.8386 ± 0.0119	0.8843 ± 0.0107	0.8921 ± 0.0084	0.9047 ± 0.0142	0.9938 ± 0.0027	25.78 ± 2.94
Ours w/o Response-difference Term	0.8428 ± 0.0086	0.8871 ± 0.0131	0.8845 ± 0.0116	0.9116 ± 0.0069	0.9943 ± 0.0030	24.36 ± 3.18
Ours w/Fixed MEA Fusion	0.8354 ± 0.0147	0.8862 ± 0.0094	0.8969 ± 0.0078	0.8992 ± 0.0128	0.9935 ± 0.0024	25.11 ± 2.41
Ours	0.8479 ± 0.0055	0.8937 ± 0.0113	0.8958 ± 0.0072	0.9141 ± 0.0034	0.9949 ± 0.0040	23.47 ± 2.46
Clinical brain tumor dataset						
SegFormer	0.7453 ± 0.0171	0.8079 ± 0.0137	0.8115 ± 0.0188	0.8426 ± 0.0200	0.9897 ± 0.0033	35.28 ± 2.02
SegFormer + ADPR	0.7708 ± 0.0116	0.8271 ± 0.0158	0.8424 ± 0.0069	0.8552 ± 0.0176	0.9909 ± 0.0042	32.64 ± 3.77
SegFormer + MEA	0.7649 ± 0.0193	0.8328 ± 0.0084	0.8295 ± 0.0161	0.8617 ± 0.0062	0.9906 ± 0.0027	31.98 ± 2.59
Ours w/Single-prototype ADPR	0.7823 ± 0.0174	0.8315 ± 0.0146	0.8468 ± 0.0181	0.8524 ± 0.0157	0.9911 ± 0.0038	31.57 ± 3.63
Ours w/o Response-difference Term	0.7761 ± 0.0205	0.8346 ± 0.0168	0.8547 ± 0.0096	0.8462 ± 0.0203	0.9907 ± 0.0029	33.29 ± 4.15
Ours w/Fixed MEA Fusion	0.7879 ± 0.0126	0.8378 ± 0.0112	0.8396 ± 0.0154	0.8649 ± 0.0089	0.9914 ± 0.0032	30.71 ± 2.86
Ours	0.7937 ± 0.0132	0.8422 ± 0.0091	0.8509 ± 0.0104	0.8673 ± 0.0130	0.9918 ± 0.0035	29.86 ± 1.62

**Table 7** presents the ablation results of ADPR and MEA on two brain tumor segmentation datasets. It can be observed that when SegFormer is used as the baseline model, it achieves relatively stable baseline performance on both the Brain Tumor Dataset and the Clinical Brain Tumor Dataset. After introducing the proposed modules, however, all metrics are further improved. On the Brain Tumor Dataset, after introducing ADPR, mIoU and mDice increase from 0.8124 and 0.8586 to 0.8326 and 0.8782, respectively, indicating that the adaptive dual-prototype representation can enhance the semantic discrimination capability of tumor regions through category-level modeling of background prototypes and tumor prototypes. After introducing MEA, mRecall reaches 0.9027 and HD95 decreases to 26.74, demonstrating that morphological evolution modeling helps capture irregular tumor regions and boundary structures. The complete model achieves the best overall performance on both datasets. Specifically, on the Brain Tumor Dataset, mIoU, mDice, and HD95 reach 0.8479, 0.8937, and 23.47, respectively, while on the Clinical Brain Tumor Dataset, they reach 0.7937, 0.8422, and 29.86, respectively. Compared with using ADPR or MEA alone, the complete model further improves region overlap accuracy and reduces boundary error. This indicates that the semantic prototype calibration of ADPR and the morphological structure enhancement of MEA are complementary, and their combination can simultaneously alleviate category confusion and boundary irregularity in brain tumor segmentation, thereby improving the segmentation stability and generalization ability of the model on both public and clinical datasets.

### 4.3 Qualitative segmentation results

**Figure 6:**
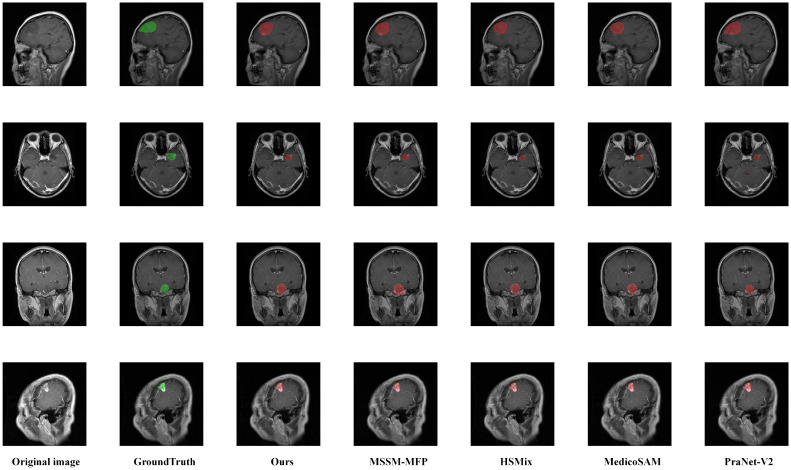
Qualitative segmentation comparison of different models on the public brain tumor dataset.

To further intuitively demonstrate the segmentation performance of the proposed method in brain tumor localization, boundary recovery, and small-structure recognition, this study selects representative comparison models, including MSSM-MFP, HSMix, MedicoSAM, and PraNet-V2, for qualitative visualization analysis, and compares their prediction results with those of the proposed method side by side. The qualitative experiments mainly focus on the ability of different models to completely cover the main tumor regions, delineate blurred boundaries, and suppress false segmentation of background tissues, thereby complementing the visual segmentation differences that cannot be fully reflected by quantitative metrics alone. By displaying the differences among the original MRI images, ground-truth masks, and prediction results of each model, the actual segmentation performance of different methods under complex brain tumor morphology can be more clearly evaluated. The experimental results on the public dataset are first shown in **Figure 6**.

**Figure 7:**
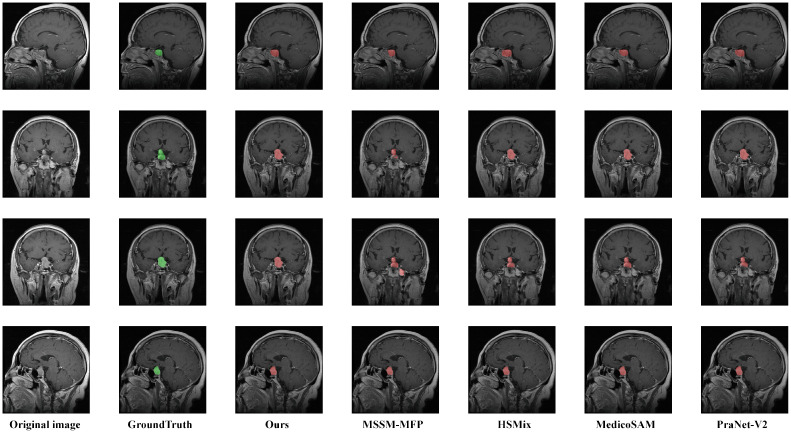
Qualitative segmentation comparison of different models on the clinical brain tumor dataset.

**Figure 6** shows the qualitative segmentation results of different models on the public Brain Tumor Dataset. It can be observed that MSSM-MFP, HSMix, MedicoSAM, and PraNet-V2 can roughly localize tumor regions, but boundary deviation, regional under-segmentation, or local false detection still occurs in some samples. These problems are particularly obvious when the tumor scale is small, the boundary contrast is low, or the tumor morphology is irregular, where there remains a certain gap between the predicted masks and the ground-truth annotations. In contrast, the segmentation results generated by the proposed method are more consistent with the ground-truth masks in terms of tumor location, main-region coverage, and boundary contour. The proposed method can better preserve the overall morphology of tumor regions and reduce erroneous responses from background tissues. This indicates that ADPR enhances the semantic discrimination capability between tumor and background through dual-prototype representation, while MEA further strengthens the morphological perception capability of irregular boundaries and local structures, enabling the model to achieve more stable and accurate visual segmentation results under complex brain tumor morphology. Furthermore, the experimental results using the clinical dataset are presented, as shown in **Figure 7**.

**Figure 7** shows the qualitative segmentation results of different models on the clinical brain tumor MRI dataset. It can be observed that tumor regions in clinical samples usually exhibit weaker boundary contrast and more obvious morphological variations, which makes MSSMMFP, HSMix, MedicoSAM, and PraNet-V2 prone to producing undersized prediction regions, discontinuous boundaries, or missing local structures in some cases. In contrast, the proposed method can more accurately cover the main tumor regions under different imaging planes and different tumor morphologies, and its predicted masks show higher consistency with the ground-truth annotations in terms of both location and shape. Especially when the tumor boundary is relatively blurred or the local structure is relatively small, the proposed method can still maintain a more complete regional response. This indicates that ADPR can enhance the semantic discrimination capability between tumor and background tissues, while MEA further improves the model’s perception capability of irregular boundaries and local structural features in clinical images, thereby achieving more stable clinical segmentation results.

### 4.4 Visualization analysis of attention response and segmentation error

**Figure 8:**
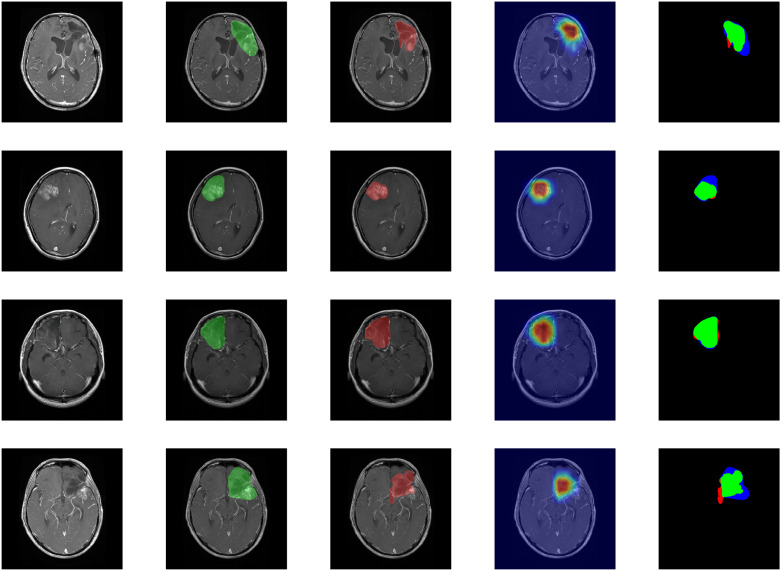
Visual interpretability and segmentation error analysis of the proposed method on the public brain tumor dataset.

To further analyze the regional attention capability and segmentation error distribution of the proposed model on the public Brain Tumor Dataset, this study conducts visual interpretability analysis on several test samples. **Figure 8** sequentially presents the original MRI image, groundtruth tumor mask, prediction result of the proposed method, model response heatmap, and prediction error visualization result. From the heatmaps, it can be observed that the high-response regions of the model are mainly concentrated around the true tumor locations, indicating that the proposed ADPR and MEA can effectively guide the network to focus on tumor-related regions rather than being disturbed by large areas of background tissue. Meanwhile, the prediction results show high consistency with the ground-truth annotations, and the error regions are mainly concentrated around tumor boundaries and local morphological transition areas, suggesting that the model has strong localization capability for the main tumor regions. These results further demonstrate that the dual-prototype semantic calibration provided by ADPR helps enhance the category response of tumor regions, while the morphological evolution mechanism introduced by MEA improves the model’s perception capability for irregular boundaries and local structures, thereby enabling the model to exhibit good interpretability and stability on the public brain tumor dataset.

### 4.5 Hyperparameter sensitivity experiment

#### 4.5.1 Sensitivity analysis of ADPR prototype number

To analyze the influence of the prototype number *K* in the ADPR module on brain tumor segmentation performance, this study conducts sensitivity experiments with different prototype number settings while keeping the network structure, training strategy, and other hyperparameters unchanged. The experiments are performed on two brain tumor segmentation datasets to evaluate the stability and adaptability of the dual-prototype representation under different data distributions. The detailed experimental results are shown in **Table 8**.

**Table 8:** Sensitivity analysis of ADPR prototype number on two brain tumor segmentation datasets.

Prototype Number K	mIoU	mDice	mPrecision	mRecall	PAcc	HD95
Figshare Brain Tumor Dataset						
2	0.8287 ± 0.0106	0.8772 ± 0.0144	0.8831 ± 0.0087	0.8978 ± 0.0176	0.9934 ± 0.0029	27.96 ± 3.38
4	0.8479 ± 0.0055	0.8937 ± 0.0113	0.8958 ± 0.0072	0.9141 ± 0.0034	0.9949 ± 0.0040	23.47 ± 2.46
6	0.8345 ± 0.0148	0.8826 ± 0.0092	0.8909 ± 0.0179	0.9023 ± 0.0125	0.9938 ± 0.0017	26.81 ± 4.22
8	0.8173 ± 0.0227	0.8654 ± 0.0186	0.8736 ± 0.0121	0.8849 ± 0.0270	0.9917 ± 0.0038	31.75 ± 5.64
10	0.8062 ± 0.0195	0.8575 ± 0.0231	0.8518 ± 0.0168	0.8816 ± 0.0204	0.9911 ± 0.0046	35.92 ± 3.17
Clinical dataset						
2	0.7664 ± 0.0185	0.8231 ± 0.0167	0.8352 ± 0.0129	0.8475 ± 0.0213	0.9902 ± 0.0037	33.97 ± 3.76
4	0.7937 ± 0.0132	0.8422 ± 0.0091	0.8509 ± 0.0104	0.8673 ± 0.0130	0.9918 ± 0.0035	29.86 ± 1.62
6	0.7812 ± 0.0169	0.8334 ± 0.0148	0.8556 ± 0.0175	0.8519 ± 0.0188	0.9909 ± 0.0020	32.18 ± 4.89
8	0.7549 ± 0.0256	0.8118 ± 0.0217	0.8170 ± 0.0202	0.8354 ± 0.0235	0.9887 ± 0.0042	39.73 ± 5.33
10	0.7423 ± 0.0208	0.7985 ± 0.0241	0.8066 ± 0.0263	0.8280 ± 0.0294	0.9876 ± 0.0048	42.91 ± 6.42

**Table 8** presents the sensitivity analysis results of the prototype number *K* in ADPR on two brain tumor segmentation datasets. It can be observed that the model performance varies with different prototype number settings, indicating that the representation capacity of category prototypes has an important influence on the semantic calibration ability of ADPR. As the number of prototypes increases from 2 to 4, the model performance continues to improve, suggesting that appropriately increasing the number of category prototypes can more comprehensively characterize the feature distribution differences within background tissues and tumor regions, thereby enhancing the category semantic representation capability of ADPR. On the Figshare Brain Tumor Dataset, when *K* = 4, the model achieves the best results in terms of mIoU, mDice, mPrecision, mRecall, PAcc, and HD95, reaching 0.8479, 0.8937, 0.8958, 0.9141, 0.9949, and 23.47, respectively. On the clinical dataset, *K* = 4 also achieves the best overall performance in terms of mIoU, mDice, mRecall, PAcc, and HD95. When *K* is further increased to 6, 8, or 10, the model performance decreases, suggesting that excessive prototypes may introduce redundant category centers, weaken the stability of prototype responses, and increase the matching noise between pixel features and category prototypes. Considering the results on both datasets, *K* = 4 achieves a good balance between prototype representation capability and model stability. Therefore, the number of prototypes for each category in ADPR is finally set to 4 in this study.

#### 4.5.2 Sensitivity analysis of MEA coefficient

**Figure 9:**
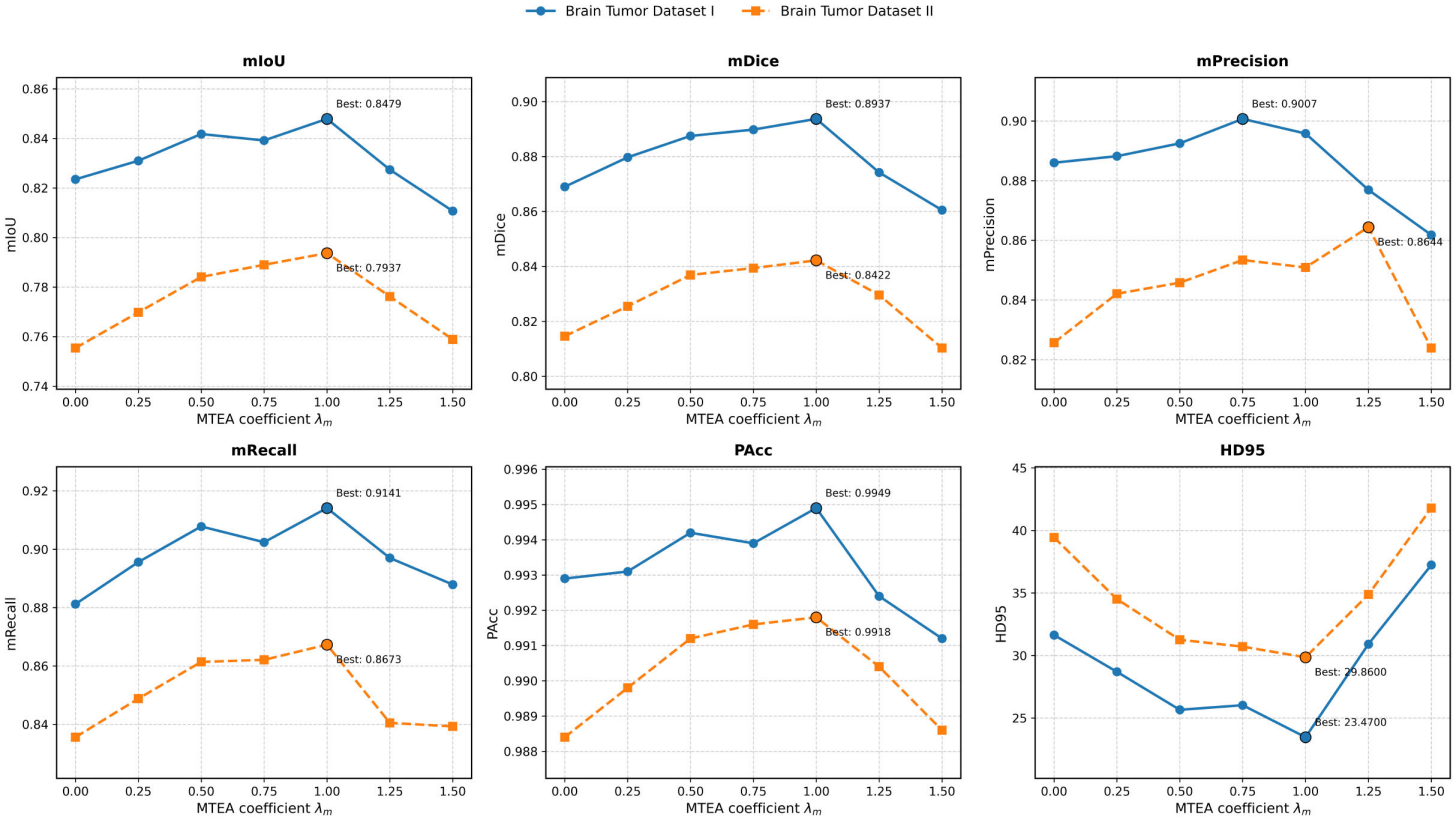
Sensitivity analysis of the MEA coefficient on two brain tumor segmentation datasets.

To further analyze the influence of the morphological evolution strength in the MEA module on brain tumor segmentation performance, this study conducts sensitivity experiments with different MEA coefficient settings while keeping the backbone network, training strategy, ADPR prototype number, and other hyperparameters unchanged. This coefficient is used to adjust the contribution of morphology-enhanced features to the overall feature representation, thereby affecting the model’s response capability to tumor boundaries, local structures, and irregular morphological regions. Specifically, *λ_m_* is defined as an external global morphological enhancement strength coefficient that controls the injection proportion of the MEA output into the final feature representation. The detailed experimental results are shown in **Figure 9**.

**Figure 9** shows the sensitivity analysis results of the MEA coefficient *λ_m_* on two brain tumor segmentation datasets. It can be observed that as *λ_m_* increases from 0.00 to 1.00, mIoU, mDice, mRecall, and PAcc on both datasets generally show an upward trend, while HD95 gradually decreases. This indicates that appropriately enhancing morphological evolution features can effectively improve the model’s ability to model tumor region completeness, boundary continuity, and local irregular structures. When *λ_m_* = 0.00, the model does not sufficiently utilize the morphological enhancement information provided by MEA, resulting in relatively limited capability in delineating blurred boundaries and morphologically complex regions. When *λ_m_* = 1.00, the model achieves favorable overall performance on both datasets, indicating that this setting can form a better balance between semantic features and morphological evolution features. When *λ_m_* is further increased to 1.25 or 1.50, most metrics decrease, while HD95 increases obviously, suggesting that excessively strong morphological enhancement may introduce oversmoothing or local structural disturbance and weaken the stable representation of original semantic features. Overall, *λ_m_* = 1.00 can fully exploit the enhancement effect of MEA on tumor boundaries and morphological structures. Therefore, the MEA coefficient is finally set to 1.00 in this study.

## 5 Discussion

Brain tumor MRI image segmentation plays an important role in clinical diagnosis and treatment workflows, as its results can provide quantitative references for lesion localization, preoperative evaluation, treatment planning, and follow-up observation. Since brain tumor regions often exhibit blurred boundaries, irregular morphology, heterogeneous local enhancement, and similar grayscale appearances to surrounding brain tissues, traditional segmentation models are prone to missed segmentation, boundary deviation, and background false positives. The dual-prototype morphological evolution network proposed in this study improves brain tumor segmentation performance from the perspectives of semantic discrimination and morphological modeling. Specifically, ADPR constructs background and tumor prototypes to enhance the model’s ability to distinguish tumor regions from normal brain tissues. MEA further strengthens the perception of irregular boundaries, local small structures, and structural continuity through differentiable morphological evolution modeling. Therefore, the proposed method can not only improve the overall overlap accuracy of tumor regions, but also reduce boundary localization errors, making the segmentation results more consistent with the clinical requirements for lesion contour completeness and boundary stability in medical imaging.

From the perspective of clinical application, the model achieves stable experimental performance on both public brain tumor datasets and clinical brain tumor MRI datasets, indicating that the proposed method has a certain degree of generalization ability under different data sources and imaging distributions. In particular, in clinical datasets, imaging acquisition conditions, scanning planes, tumor sizes, tissue contrast, and boundary clarity may vary substantially, which imposes higher requirements on the robustness of segmentation models. The experimental results show that the proposed method achieves superior performance in terms of mIoU, mDice, mRecall, PAcc, and HD95, indicating that it can reduce boundary deviation and missed segmentation risks while maintaining sufficient coverage of the main tumor regions. For clinical assistance scenarios, such stable region identification and boundary restoration capabilities may help improve the consistency of brain tumor image analysis and provide a more reliable segmentation basis for subsequent volume measurement, lesion change assessment, and treatment response evaluation. However, the proposed method should still be regarded as a clinical auxiliary analysis tool, and its outputs should be comprehensively interpreted together with the professional judgment of radiologists and neurosurgeons rather than replacing clinical diagnosis and treatment decision-making.

Although the proposed method achieves favorable experimental results, several limitations remain. Failure cases are mainly observed in samples with extremely low tumor–background contrast, very small lesion regions, or highly irregular boundary transitions, where the model may still produce partial boundary deviations or local under-segmentation. In addition, when surrounding brain tissues exhibit enhancement patterns or grayscale distributions similar to tumor regions, the model may generate limited false-positive responses in adjacent non-tumor areas. First, this study mainly focuses on the binary brain tumor segmentation task, which only distinguishes tumor regions from background regions, without further separating fine-grained internal structures such as necrotic regions, edema regions, and enhancing regions, or directly performing joint segmentation and classification of different tumor types. Second, the current experiments are mainly based on two-dimensional MRI slices. Although this setting reduces computational complexity and facilitates model training, the utilization of three-dimensional spatial continuity and inter-slice contextual relationships remains limited. Third, the scale and sources of the clinical data still need to be further expanded. In the current experimental design, the public Figshare dataset and the clinical dataset were organized as two separate data sources. The public dataset was divided into training, validation, and testing subsets at the patient level, and its validation subset was used for hyperparameter and checkpoint selection. The clinical dataset was retrospectively collected from routine clinical examinations and was further divided into training and testing subsets at the case level. Therefore, the clinical experiment should be regarded as a case-level evaluation on an independent testing subset from the same retrospective clinical cohort, rather than a strict external validation based on a completely independent external center. Moreover, because the clinical dataset was retrospectively collected from routine examinations, some acquisition-related metadata were incomplete, which may limit a more fine-grained analysis of cross-device and cross-parameter generalization. Accordingly, the current clinical results provide preliminary evidence for the applicability of the proposed method to clinical MRI data, but the generalizability of the model across independent institutions, scanners, imaging protocols, and patient populations still requires further validation. In addition, the current clinical-oriented interpretation is still based on segmentation performance and visual analysis, while reader studies, uncertainty estimation, quantitative attention-mask overlap analysis, and more detailed clinical workflow validation have not yet been included. Future studies should conduct external validation across more centers, scanning devices, and MRI sequence conditions to further evaluate the stability and applicability of the model in real clinical environments. In addition, this study has not yet systematically analyzed the practical performance of the model in terms of inference speed, memory consumption, and clinical system deployment. Future research may further integrate multimodal MRI sequences, threedimensional segmentation networks, semi-supervised learning, and uncertainty estimation methods to improve the fine-grained recognition capability for complex brain tumor structures and promote interpretable, safe, and reliable application in clinical auxiliary diagnosis and treatment workflows.

## 6 Conclusion

This study proposes a dual-prototype morphological evolution network for binary segmentation of brain tumor MRI images. The proposed method adopts SegFormer as the backbone network and constructs category prototypes for background and tumor regions through the adaptive dualprototype representation module, thereby enhancing the model’s ability to distinguish semantic differences between normal brain tissues and tumor tissues. Meanwhile, the morphological evolution attention module introduces a differentiable morphological modeling mechanism, in which soft dilation, soft erosion, soft opening, and soft closing operations are used to characterize boundary variations, local structures, and morphological continuity of tumor regions. Experimental results demonstrate that the proposed method achieves superior segmentation performance on both the public brain tumor dataset and the clinical brain tumor MRI dataset, outperforming or achieving competitive performance compared with several mainstream segmentation methods in terms of region overlap, pixel-level recognition, and boundary localization. Ablation experiments and visualization results further verify the effectiveness and complementarity of ADPR and MEA, indicating that semantic prototype calibration and morphological structure enhancement can jointly improve the accuracy, stability, and clinical applicability potential of brain tumor segmentation. From a clinical perspective, segmentation errors may affect downstream tasks such as tumor volume measurement, surgical planning, radiotherapy target delineation, and treatment response assessment. Therefore, reducing boundary deviation, missed segmentation, and false-positive regions is important for improving the reliability of brain tumor image-assisted analysis.

Future research will further extend this work from the perspectives of data, model design, and clinical application. First, multi-center clinical data with more sources, larger scales, and more MRI sequences can be introduced to further validate the generalization ability of the model under different scanning devices, imaging protocols, and tumor types. Second, the current two-dimensional slice-based segmentation framework can be extended to three-dimensional brain tumor segmentation tasks to fully exploit inter-slice spatial continuity and volumetric contextual information, thereby improving the modeling capability for complex tumor structures and subtle boundary variations. In addition, future studies may further integrate uncertainty estimation, semi-supervised learning, and multimodal fusion strategies to enhance the reliability of the model under limited annotation conditions and complex clinical scenarios.More clinically oriented evaluation, such as reader studies, volume consistency analysis, and radiotherapy target-related assessment, may also be further conducted to clarify how segmentation quality influences practical clinical workflows. With continuous optimization of model interpretability, inference efficiency, and clinical deployment workflows, the proposed method is expected to provide more stable technical support for brain tumor image-assisted analysis, lesion quantitative assessment, and treatment follow-up.

## Data Availability

The original contributions presented in the study are included in the article/supplementary material. Further inquiries can be directed to the corresponding author.

## Appendix

Illustrative visualization of ADPR prototype response maps. Each row presents one representative brain tumor MRI case, including the original image, ground-truth tumor mask, background prototype response, and tumor prototype response. The background response maps show stronger activation in non-tumor brain tissues with locally suppressed responses around the tumor regions, whereas the tumor response maps exhibit relatively higher activation around the annotated lesion areas while still retaining weak responses and noise in surrounding tissues. These visualization results illustrate the semantic separation tendency between background-oriented and tumor-oriented prototype responses.

## References

[1] Khan MKH Guo W Liu J Dong F Li Z Patterson TA . Machine learning and deep learning for brain tumor mri image segmentation. Exp Biol Med. (2023) 248:1974–92. doi: 10.1177/15353702231214259 38102956 PMC10798183

[2] Dorfner FJ Patel JB Kalpathy-Cramer J Gerstner ER Bridge CP . A review of deep learning for brain tumor analysis in mri. NPJ Precis Oncol. (2025) 9:2. doi: 10.1038/s41698-024-00789-2 39753730 PMC11698745

[3] Qiao S Guo Q He X Wang M Wang S Singh AK . Rldj-w: a reinforcement learning-driven joint watermarking framework for privacy leakage detection in digital healthcare systems. IEEE Internet Things J. (2025).

[4] Ahamed MF Hossain MM Nahiduzzaman M Islam MR Islam MR Ahsan M . A review on brain tumor segmentation based on deep learning methods with federated learning techniques. Comput Med Imaging Graphics. (2023) 110:102313. doi: 10.1016/j.compmedimag.2023.102313 38011781

[5] Abidin ZU Naqvi RA Haider A Kim HS Jeong D Lee SW . Recent deep learning-based brain tumor segmentation models using multi-modality magnetic resonance imaging: a prospective survey. Front Bioeng Biotechnol. (2024) 12:1392807. doi: 10.3389/fbioe.2024.1392807 39104626 PMC11298476

[6] Lee J Shin D Oh S-H Kim H . Method to minimize the errors of ai: quantifying and exploiting uncertainty of deep learning in brain tumor segmentation. Sensors. (2022) 22:2406. doi: 10.3390/s22062406 35336577 PMC8951581

[7] Yang Q Guo X Chen Z Woo PY Yuan Y . D 2-net: dual disentanglement network for brain tumor segmentation with missing modalities. IEEE Trans Med Imaging. (2022) 41:2953–64. doi: 10.1109/tmi.2022.3175478 35576425

[8] Liu Y Mu F Shi Y Cheng J Li C Chen X . Brain tumor segmentation in multimodal mri via pixel-level and feature-level image fusion. Front Neurosci. (2022) 16:1000587. doi: 10.3389/fnins.2022.1000587 36188482 PMC9515796

[9] Yang H Zhou T Zhou Y Zhang Y Fu H . Flexible fusion network for multi-modal brain tumor segmentation. IEEE J BioMed Health Inf. (2023) 27:3349–59. doi: 10.1109/jbhi.2023.3271808 37126623

[10] Jiang Y Zhang Y Lin X Dong J Cheng T Liang J . Swinbts: a method for 3d multimodal brain tumor segmentation using swin transformer. Brain Sci. (2022) 12:797. doi: 10.3390/brainsci12060797 35741682 PMC9221215

[11] Cao Y Zhou W Zang M An D Feng Y Yu B . Mbanet: a 3d convolutional neural network with multi-branch attention for brain tumor segmentation from mri images. BioMed Signal Process Control. (2023) 80:104296. doi: 10.1016/j.bspc.2022.104296 38826717

[12] Ting H Liu M . Multimodal transformer of incomplete mri data for brain tumor segmentation. IEEE J BioMed Health Inf. (2023) 28:89–99. doi: 10.1109/jbhi.2023.3286689 37327094

[13] Liu H Huang J Li Q Guan X Tseng M . A deep convolutional neural network for the automatic segmentation of glioblastoma brain tumor: joint spatial pyramid module and attention mechanism network. Artif Intell Med. (2024) 148:102776. doi: 10.1016/j.artmed.2024.102776 38325925

[14] Yadav AC Kolekar MH Sonawane Y Kadam G Tiwarekar S Kalbande DR . Effunet++: a novel architecture for brain tumor segmentation using flair mri images. IEEE Access. (2024) 12:152430–43. doi: 10.1109/access.2024.3480271 25079929

[15] Aslam W Hussain J Aslam MZ Jan S Riaz TB Iqbal A . Enhanced brain tumor segmentation in medical imaging using multi-modal multi-scale contextual aggregation and attention fusion. Sci Rep. (2025) 15:37308. doi: 10.1038/s41598-025-21255-4 41136620 PMC12552493

[16] Zhang M Sun Q Han Y Zhang M Wang W Zhang J . Generative adversarial dacformer network for mri brain tumor segmentation: m. zhang et al. Sci Rep. (2025) 15:17840. doi: 10.1038/s41598-025-02714-4 40404794 PMC12098717

[17] Jin C Noor NSEM Ng TF Asaari MSM Ibrahim H . Transformer-based architectures in mri brain tumor segmentation: a review. Comput Med Imaging Graphics. (2026), 102729. doi: 10.1016/j.compmedimag.2026.102729 41723899

[18] Rezk AM Al-Fakih A Shazly A Singh VK Roh YH Ryu K . Regionalaware and sequence-informed multi-decoder network for robust brain glioma segmentation in multi-parametric mri. Comput Biol Med. (2026) 201:111387. doi: 10.1016/j.compbiomed.2025.111387 41411810

[19] Ronneberger O Fischer P Brox T . U-net: convolutional networks for biomedical image segmentation. In: International Conference on Medical image computing and computerassisted intervention. Cham: Springer (2015). p. 234–41.

[20] Isensee F Jaeger PF Kohl SA Petersen J Maier-Hein KH . nnu-net: a self-configuring method for deep learning-based biomedical image segmentation. Nat Methods. (2021) 18:203–11. doi: 10.1038/s41592-020-01008-z 33288961

[21] Chen L-C Zhu Y Papandreou G Schroff F Adam H . (2018). “ Encoder-decoder with atrous separable convolution for semantic image segmentation”, in: Proceedings of the European conference on computer vision (ECCV) (Cham: Springer), 801–18.

[22] Xie E Wang W Yu Z Anandkumar A Alvarez JM Luo P . Segformer: simple and efficient design for semantic segmentation with transformers. Adv Neural Inf Process Syst. (2021) 34:12077–90.

[23] Pan Y Zhang S Gernand AD Goldstein JA Wang JZ . S2s2: semantic stacking for robust semantic segmentation in medical imaging. Proc AAAI Conf Artif Intell. (2025) 39:6335–44. 10.1609/aaai.v39i6.32678 PMC13035368 41919077

[24] Zhang P Dong Y Li J Jiang L Hu M Ping Y . Mssm-mfp: medical semantic segmentation model based on multiscale fusion perception. BioMed Signal Process Control. (2026) 112:108481. doi: 10.1016/j.bspc.2025.108481 38826717

[25] Dong X Wang L Lv X Zhang X Zhang H Pu B . Certaintta: estimating uncertainty for test-time adaptation on medical image segmentation. Inf Fusion. (2025) 123:103300. doi: 10.1016/j.inffus.2025.103300 38826717

[26] Cheng B Misra I Schwing AG Kirillov A Girdhar R . (2022). “ Masked-attention mask transformer for universal image segmentation”, in: Proceedings of the IEEE/CVF conference on computer vision and pattern recognition (Piscataway: IEEE), 1290–9.

[27] Archit A Freckmann L Pape C . Medicosam: robust improvement of sam for medical imaging. IEEE Trans Med Imaging. (2025). doi: 10.1109/tmi.2025.3644811 41406266

[28] Sun D Dornaika F Barrena N . Hsmix: hard and soft mixing data augmentation for medical image segmentation. Inf Fusion. (2025) 115:102741. doi: 10.1016/j.inffus.2024.102741 38826717

[29] Hu B-C Ji G-P Shao D Fan D-P . Pranet-v2: dual-supervised reverse attention for medical image segmentation. Comput Visual Media. (2026). doi: 10.26599/cvm.2025.9450510

